# Supplementary material for: Crawling and Gliding: A Computational Model for Shape-Driven Cell Migration
Source: PLoS Comput Biol. 2015 Oct 21;11(10):e1004280. doi: 10.1371/journal.pcbi.1004280 (PMC4619082; doi:10.1371/journal.pcbi.1004280)
Supplement: S1 Code — (ZIP) [file pcbi.1004280.s012.zip › release/tst/doc/html/classInfo.html]

Tissue Simulation Toolkit: Info Class Reference


Tissue Simulation Toolkit
 0.1.4.1


- Main Page
- Namespaces
- Classes
- Files

- Class List
- Class Hierarchy
- Class Members

Public Member Functions |
List of all members

Info Class Reference

Enables interactive querying of the simulation.
More...

`#include <info.h>`

|  |  |
| --- | --- |
| Public Member Functions | |
|  | Info (Dish &dish, Graphics &graphics, std::ostream &out=std::cout) |
|  | Constructs and Info class dish with specified graphics window. More... |
|  | |
| void | Menu (void) |
|  | Reads out key presses in the Graphics window and interprets them. More... |
|  | |
| void | WriteCOM (int cell\_id, std::ostream &out=std::cout) |
|  | Writes center of mass of cell "cell\_id" to stream out. More... |
|  | |
| Cell & | ClickCell (Graphics \*g) |
|  | Waits until the user clicks a cell and returns a reference to it. More... |
|  | |

## Detailed Description

Enables interactive querying of the simulation.

Only using key presses, to be defined in Info::Menu().

Right-click menu in future Qt-linked versions?

## Constructor & Destructor Documentation

|  |  |  |  |
| --- | --- | --- | --- |
| Info::Info | ( | Dish & | *dish*, |
|  |  | Graphics & | *graphics*, |
|  |  | std::ostream & | *out* = `std::cout` |
|  | ) |  |  |

Constructs and Info class dish with specified graphics window.

Parameters
:   |  |  |
    | --- | --- |
    | dish | The virtual Petri dish to query. |
    | graphics | The Graphics window displaying the dish. |
    | out | (optional) Stream into which info is written. Default: console. |

References Info().

Referenced by Info().

## Member Function Documentation

|  |  |  |  |  |  |
| --- | --- | --- | --- | --- | --- |
| Cell & Info::ClickCell | ( | Graphics \* | *g* | ) |  |

Waits until the user clicks a cell and returns a reference to it.

References Graphics::GetXYCoo().

|  |  |  |  |  |  |
| --- | --- | --- | --- | --- | --- |
| void Info::Menu | ( | void |  | ) |  |

Reads out key presses in the Graphics window and interprets them.

If you want to define extra interactive queries, redefine this method.

If you want a nice GUI menu, reimplement this method.

References Cell::Area(), Cell::Colour(), Parameter::lambda, Cell::MeasureCellSize(), par, ReadDouble(), Cell::SetTargetArea(), Cell::SetTargetLength(), Cell::setTau(), Cell::Sigma(), Parameter::size\_init\_cells, Cell::TargetArea(), and YesNoP().

|  |  |  |  |
| --- | --- | --- | --- |
| void Info::WriteCOM | ( | int | *cell\_id*, |
|  |  | std::ostream & | *out* = `std::cout` |
|  | ) |  |  |

Writes center of mass of cell "cell\_id" to stream out.

---

The documentation for this class was generated from the following files:

- info.h
- info.cpp


---

Generated on Thu Aug 14 2014 22:04:01 for Tissue Simulation Toolkit by  

 1.8.6
